# Sociodemographic and Occupational Factors Associated With Burnout: A Study Among Frontline Healthcare Workers During the COVID-19 Pandemic

**DOI:** 10.3389/fpubh.2022.854687

**Published:** 2022-03-09

**Authors:** Duaa Aljabri, Fatimah Alshatti, Arwa Alumran, Saja Al-Rayes, Deema Alsalman, Arwa Althumairi, Nouf Al-kahtani, Mohammad Aljabri, Shaheed Alsuhaibani, Turki Alanzi

**Affiliations:** ^1^Department of Health Information Management and Technology, College of Public Health, Imam Abdulrahman Bin Faisal University, Dammam, Saudi Arabia; ^2^General Administration of Medical Consultations, Ministry of Health, Riyadh, Saudi Arabia; ^3^Department of Urology, College of Medicine, King Fahd University Hospital, Imam Abdulrahman Bin Faisal University, Dammam, Saudi Arabia

**Keywords:** Copenhagen Burnout Inventory (CBI), burnout, healthcare workers, sociodemographic factors, occupational health

## Abstract

**Purpose:**

To describe the prevalence of burnout among frontline healthcare workers (HCWs) during the COVID-19 pandemic and the associated sociodemographic and occupational factors.

**Methods:**

A cross sectional survey study was carried out to study HCWs burnout using the 19-item Full Copenhagen Burnout Inventory (CBI) that includes personal, work, and patient-related burnout subscales. Bivariate analysis was used to test for associations and *p* < 0.05 was considered statistically significant.

**Results:**

A total of 207 responses received; where the mean score of personal burnout was 67.23, the mean of work-related burnout was 61.38, and the mean of patient-related burnout was 54.55. Significant associations were found; where female HCWs, those working in rotating day-and-night shifts, working more than 55-h per week, and who had their shift time and hours changed during the pandemic, had higher levels of personal and work-related burnout (*P* < 0.05). Patient-related burnout was higher among those who were single (divorced or separated), nurses, non-Citizens, those with fewer years of experience, and who were infected by COVID-19 and have been quarantined (*P* < 0.05). Age was not a significant factor of burnout in any of the CBI subscales.

**Conclusions:**

There is a prevalent level of burnout among frontline HCWs during the COVID-19 pandemic. Findings highlight key sociodemographic and occupational factors affecting burnout; which can help planning for psychological support strategies. Furthermore, effective administrative control is important to institute policies and mechanisms to identify, and freely report burnout symptoms among HCWs to promote their wellbeing.

## Introduction

Burnout is defined as a psychological disorder characterized by an adverse emotional reaction to a job resulting from working in a stressful environment (1–3). Healthcare is perceived as one of the most stressful working environments as it requires intensive personal interactions with patients and other healthcare workers (HCWs) (4). Thus, among HCWs, burnout is a well-known severe problem that has received increased attention in recent years (5). Former studies have reported that HCWs can experience anxiety and depression symptoms due to stressful working conditions and high volumes of work, which consequently develops negative outcomes such as burnout (6, 7). Among studies that investigated the association between burnout and working in healthcare settings, burnout was a significant problem in excess-work settings such as emergency and critical care (5, 8).

The increased attention to HCWs burnout is driven by its negative consequences on patient safety, consistency of care, health system costs and workflow, and HCWs own safety and care (9, 10). Further, many studies confirmed that a significant relationship exists between the risk of medical errors and burnout (11–13). Shanafelt et al. (8) reported that a high number of major medical errors has been significantly associated with burnout among physicians. In addition, a significant association was reported between high burnout and therapeutic and diagnostic errors (13). Medical errors among physicians with either a few or significant burnout symptoms are higher compared with physicians who do not have burnout symptoms (14). The risk among nurses should not be underestimated, as burnout can impact their productivity and reduce the quality of care provided, especially with them being at higher risk of insomnia and sleep disturbance (15, 16). Thus, the burnout effect is not limited to losses among humans but also the whole healthcare system (17).

Not long after COVID-19 emerged in China, in late 2019, the World Health Organization (WHO) considered it a pandemic, a severe problem endangering human health (18). The first case reported in Saudi Arabia was on March 2nd, 2020. The spread of COVID-19 infection has created higher demands on the global healthcare systems and frontline HCWs have been playing an instrumental role by providing the necessary care for confirmed or suspected COVID-19 patients during the pandemic (19). Literature from previous pandemics, such as Middle East Respiratory Syndrome (MERS) and Severe Acute Respiratory Syndrome (SARS) epidemics, showed a wide range of psychological morbidities experienced by HCWs, including trauma and burnout that might last for months after the pandemic (20, 21). In addition, traumatic life events are highly related to suicidal ideation in stressful work environments (22). Perceptions of “infection stigma” from the community and social isolation also contribute to psychological distress (23). Yet, although frontline HCWs anxieties during outbreaks can lead to absence and high turnover rates, there is suggested evidence that HCWs feel a strong professional commitment and obligation to continue providing healthcare services (24). In addition, the pressure that HCWs feel to maintain high-quality healthcare during a pandemic might relate strongly to presenteeism, which is being physically present but work insufficiently because of illness (25).

There are emerging studies around the world exploring the prevalence of burnout, stress, and anxiety disorders during the COVID-19 pandemic (26, 27). A systematic review reported that the prevalence of burnout can differ between geographical regions. In Iran, over half of HCWs had high levels of burnout (28). Similarly, frontline HCWs in Italy had higher levels of emotional exhaustion than non-pandemic periods (29). On the contrary, Central Asia reported the lowest burnout symptoms (30, 31). A recent study in SA used the Maslach Burnout Inventory (MBI) to assess burnout prevalence and risk factors among HCWs. On the MBI, 38.5% of HCWs showed high emotional exhaustion, 31.2% showed high depersonalization, and 33.6% showed reduced personal achievement. The high burnout in this study was due to direct contact with infected cases and changes in the working patterns during the pandemic. Another study in SA investigated the prevalence of burnout among urologists using the Copenhagen Burnout Inventory (CBI), and found the mean personal burnout as 57.92, while the mean work-related burnout was 55.26 (32).

To fulfill high quality of care for patients and mitigate negative outcomes, there is a further a need to address healthcare workers' burnout. This study utilized the Copenhagen Burnout Inventory instead of the most common Maslach Burnout Inventory scale to assess burnout among a national sample of frontline HCWs during the COVID-19 pandemic. As the MBI considers burnout as a syndrome consisting of emotional exhaustion, depersonalization, and reduced personal accomplishment; this definition has been criticized as exhaustion alone is believed to be the core definition of burnout (33). Thus, CBI, focuses primarily on exhaustion in personal, work-related, and client-related scales (34). Furthermore, the study attempts to identify the main factors associated with the increasing burnout symptoms among frontline HCWs in Saudi Arabia.

## Methods

### Study Design and Sample

This is a cross-sectional descriptive study involving all frontline HCWs who were in direct contact with COVID-19 patients during the pandemic in Saudi Arabia. This includes physicians, nurses, pharmacists, respiratory therapists, other allied health professionals, and administrative staff.

### Data Collection

Quantitative data were collected through an anonymous self-administered questionnaire broadcasted via WhatsApp and Twitter during October 2020. Voluntary and anonymous participation was sought to complete the online survey. The invitation message included a message that explains the purpose of the study, confirmation of confidentiality of all personal information, and the study principal investigator contact details. Consent was assured by selecting “*I consent to participate in the study and have the data published in journal article”* before starting the survey.

### Study Instrument

The study questionnaire was distributed in English language as it's the official language of communication among healthcare workers. Data was collected in four sections: The first section included HCWs demographics and professional characteristics such as age, gender, nationality, HCW type, and years of experience. The second section included HCWs personal experiences with COVID-19, such as whether they have been infected with COVID-19 or quarantined due to a suspicion of being infected. The third section included HCWs work-related experiences, such as changes in their work hours, and shift times during the pandemic and their use of personal protective equipment (PPE). The fourth section included the full 19-item Copenhagen Burnout Inventory (CBI), which is a standardized scale for measuring burnout with good reliability and validity and is widely used in social science research (34, 35).

The CBI uses three subscales: personal burnout, work-related burnout, and client-related burnout. Personal burnout scale measures the participant's degree of physical and psychological exhaustion, and includes six items, such as “How often do you feel tired?” and “How often do you feel worn out?” The work-related burnout scale measures the extent of the participant's physical and psychological exhaustion with their workplace, and includes seven items, such as “Do you feel burnt out because of your work?” and “Do you feel worn out at the end of the workday?.” The client, i.e., patient-related burnout scale measures the extent of the participant's physical and psychological exhaustion with their patients, and includes six items, such as “Do you find it hard to work with patients?” and “Are you tired of working with patients?.” The scale's response options were “always,” “often,” “sometimes,” “seldom,” and “never.” The responses were converted to scores ranging from 0 to 100 (*always* = 100, *often* = 75, *sometimes* = 50, *seldom* = 25, *never* = 0) according to the previously published CBI scoring system; where higher scores means a high level of burnout (35). It is important to note that one question in the work-related scale was negatively worded “Do you have enough energy for family and friends during leisure time?,” and thus, scored reversely.

### Instrument Validity and Reliability

Several studies have provided considerable evidence to support the CBI's validity in terms of its content, internal structure (i.e., internal consistency), response process (i.e., clarity and ease of understanding), and relation to other aspects such as vitality and mental health (36). In this study, a pilot study among 15 frontline HCWs was conducted and revealed satisfactory validity levels. Internal consistency of the CBI subscales was assessed in this study with Cronbach's alpha coefficient and ranged between 0.86 and 0.90, suggesting high internal consistency.

### Statistical Analysis

For analysis, data were analyzed using IBM SPSS^®^ Statistics. Categorical data were summarized using frequency and proportions, and mean scores with standard deviations (SDs) were calculated for each burnout subscale: personal, work-related, and patient-related domains using the 0- to 100-point scale. To explore sociodemographic and occupational factors associated with each burnout domain, bivariate analysis was used using *t*-test and one-way ANOVA, as appropriate, and *p* < 0.05 was considered statistically significant.

## Results

Responses were received from 207 HCWs. All respondents were over the age of 21 years. Most HCWs were female (55%) and between 36 years and 40 years of age (35%). The majority were nurses (31%), followed by physicians (24%) and other allied workers (29%). Most respondents were working in full-time shifts (88%). Nearly 34% tested positive for COVID-19 and 83% felt at risk of being infected (Table 1).

**Table 1:** Characteristics of study participants.

**Characteristics**	***n* (%)**
**Gender**	
Male	91 (43)
Female	116 (55)
**Age in years**	
25–30	20 (9.6)
31–35	38 (18.2)
36–40	73 (34.9)
41–45	38 (18.2)
46–50	19 (9.1)
50+	19 (9.1)
**Marital status**	
Single	38 (18.2)
Married	129 (61.7)
Divorced/separated	30 (14.4)
Widowed	10 (4.8)
**Nationality**	
Saudi	174 (83.3)
Non-Saudi	33 (15.8)
**Profession**	
Physician	50 (23.9)
Nurse	65 (31.1)
Allied health professional	61 (29.2)
Administration	17 (8.1)
Other	14 (6.7)
**Years of experience**	
1–5	22 (10.5)
5–10	62 (29.7)
10–15	70 (33.5)
16+	51 (24.4)
**Work status**	
Full-time	185 (88.5)
Part-time	16 (7.7)
**Work shift**	
Day shift	87 (41.6)
Night shift	26 (12.4)
Rotating day and night	93 (44.5)
**Work hours per week**	
Less than 45	40 (19.1)
45–55	128 (61.2)
56–65	26 (12.4)
More than 65	12 (5.7)

### Personal Burnout

Around 41% reported that they “often feel worn out,” while 39% “often feel tired.” Also, 31.4% reported always feel physically exhausted and 30.4% always feel emotionally exhausted. Furthermore, 34.8% often think that they can't take it anymore (Table 2).

**Table 2:** Personal, work, and patient-related burnout among study participants.

**Questions**	**Always (%)**	**Often (%)**	**Sometimes (%)**	**Seldom (%)**	**Never (%)**
**Personal burnout (α** **=** **0.89)**					
1.How often do you feel tired?	33.3	39.1	21.3	1.9	4.3
2.How often are you physically exhausted?	31.4	31.4	30.0	5.3	1.9
3.How often are you emotionally exhausted?	30.4	30.4	27.1	9.2	2.9
4.How often do you think: “I cannot take it anymore”?	18.4	34.8	25.1	15.0	6.8
5.How often do you feel worn out?	18.8	41.1	23.7	10.1	6.3
6.How often do you feel weak and susceptible to illness?	23.2	34.3	26.1	10.1	6.3
**Work-related burnout (α** **=** **0.86)**					
7.Do you feel worn out at the end of the working day?	24.8	41.7	20.9	8.7	3.9
8.Are you exhausted in the morning at the thought of another day at work?	17.0	38.3	22.8	11.7	10.2
9.Do you feel that every working hour is tiring for you?	15.5	41.5	21.7	13.0	8.2
10.Do you have enough energy for family and friends during leisure time?	5.8	15.0	34.3	35.7	9.2
11.Is your work emotionally exhausting?	22.0	36.6	28.8	8.8	3.9
12.Does your work frustrate you?	13.5	41.5	24.6	11.6	8.7
13.Do you feel burnt out because of your work?	12.6	39.6	29.0	9.7	9.2
**Patient-related burnout (α** **=** **0.90)**					
14.Do you find it hard to work with patients?	14.5	36.2	28.0	12.1	9.2
15.Does it drain your energy to work with patients?	11.6	33.8	32.9	15.9	5.8
16.Do you find it frustrating to work with patients?	9.8	30.2	30.2	17.1	12.7
17.Do you feel that you give more than you get back when you work with patients?	11.3	32.4	32.4	14.2	9.8
18. Are you tired of working with patients?	7.7	30.0	31.4	18.4	12.6
19.Do you sometimes wonder how long you will be able to continue working with patients?	12.6	26.6	34.3	15.9	10.6

### Work-Related Burnout

Among study participants, 41.7% felt they are worn out at the end of the working day. Additionally, 41.5% feel that every working hour is tiring, 41.5% feel frustrated and 36.6% feel emotionally exhausted because of their work. Around 35.7% reported that they seldom have the energy for family and friends during leisure time (Table 2).

### Patient-Related Burnout

Concerning patient-related burnout, 36.2% often find it hard to work with patients and 33.8% reported that working with patients drain their energy. Around 30% are often tired of working with patients and 34.3% sometimes wonder how long they will be able to continue working with them (Table 2).

As presented in Table 3, the majority of participants reported considerable burnout on at least one of the CBI subscales. The mean personal burnout was 67.23, while the mean work-related burnout was 61.38, and the mean patient-related burnout was 54.55. Associations were found between the study variables and burnout subscales, where female HCWs, those working in rotating day and night shifts, those working more than 55 h per week, and those who had their shift time and shift hours changed due to the pandemic, had significantly higher burnout scores in both the personal and work-related domains (*P* < 0.05). In addition, personal burnout was significantly higher among allied health professionals, and those wearing adequate PPE while interacting with COVID-19 patients; while work burnout was higher among nurses (*P* < 0.05) (Supplementary Table 1). Patient-related burnout was the highest among divorced or separated HCWs, nurses, non-Saudi citizens, those with less years of experience, and those who have been tested positive for COVID- and quarantined (*P* < 0.05). Age was not a significant factor of burnout in any of the CBI sub-scales.

**Table 3:** Association between study variables and CBI subscales.

**Variable**	***n* (%)**	**Personal burnout**		**Work-related burnout**		**Patient-related burnout**	
		***M* (*SD*)**	**Sig. (*p*-value)**	***M* (*SD*)**	**Sig. (*p*-value)**	***M* (*SD*)**	**Sig. (*p*-value)**
**All Study Sample**	207 (100%)	67.23 (21.66)	-	61.38 (21.6)	-	54.55 (23.4)	-
**Gender**							
Male	91 (43)	62.450 (23.01)	***t** **=*** **−2.85**	55.55 (21.79)	***t*** **=** **−2.63**	53.20 (23.83)	*t* = −0.73
Female	116 (55)	70.97 (19.85)	**(<0.001)^a^**	62.95 (18.51)	**(0.02)^a^**	55.61 (23.12)	(0.38)^a^
**Age in years**							
25–30	20 (9.6)	61.87 (20.64)	*f* = 1.79 (0.12)^**b**^	58.75 (19.44)	*f* = 1.23 (0.34)^**b**^	62.08 (24.02)	*f* = 1.27 (0.47)^**b**^
31–35	38 (18.2)	70.83 (20.08)		62.51 (20.62)		56.79 (20.76)	
36–40	73 (34.9)	70.43 (19.97)		62.80 (19.12)		55.63 (22.29)	
41–45	38 (18.2)	67.10 (23.00)		56.20 (20.75)		48.22 (25.36)	
46–50	19 (9.1)	64.25 (25.77)		57.14 (20.92)		55.83 (25.47)	
50+	19 (9.1)	56.57 (22.70)		52.72 (22.74)		49.34 (25.01)	
**Marital status**							
Single	38 (18.2)	73.02 (21.41)	*f* = 1.66	61.56 (19.39)	*f* = 0.68	57.23 (26.57)	***f*** **=** **2.92**
Married	129 (61.7)	65.18 (21.08)	(0.15)^**b**^	58.46 (20.85)	(0.37)^**b**^	51.41 (21.76)	**(0.02)^b^**
Divorced/separated	30 (14.4)	70.27 (19.86)		63.57 (17.17)		64.77 (20.71)	
Widowed	10 (4.8)	62.50 (31.73)		56.96 (25.64)		54.16 (31.67)	
**Nationality**							
Saudi	174 (83.3)	67.02 (21.09)	*t* = −0.31 (0.51)^a^	58.94 (20.29)	*t* = −1.23 (0.08)^a^	52.95 (23.38)	***t*** **=** **−2.28 (0.01)^a^**
Non-Saudi	33 (15.8)	68.30 (24.78)		63.69 (20.18)		63.00 (22.01)	
**Profession**							
Physician	50 (23.9)	62.33 (23.32)		56.35 (22.40)		53.833 (23.71)	
Nurse	65 (31.1)	70.76 (20.63)	***f*** **=** **7.60 (<0.001)^b^**	65.14 (19.77)	***f*** **=** **6.22 (<0.001)^b^**	60.94 (20.80)	***f*** **=** **2.29 (0.05)^b^**
Allied health	61 (29.2)	74.93 (16.83)		63.64 (14.52)		52.24 (24.41)	
Administration	17 (8.1)	49.50 (23.65)		46.21 (23.09)		45.49(23.91)	
Other	14 (6.7)	56.25 (17.95)		45.57 (19.20)		48.51 (24.54)	
**Years of experience**							
1–5	22 (10.5)	61.17 (20.420)	*f* = 0.85 (0.38)^**b**^	54.6 (21.14)	*f* = 0.63 (0.59)^**b**^	61.55 (24.42)	***f*** **=** **3.49 (0.04)^b^**
5–10	62 (29.7)	69.620 (19.44)		61.46 (19.217)		60.71 (19.62)	
10–15	70 (33.5)	67.91 (23.37)		60.28 (21.08)		49.57 (23.45)	
16+	51 (24.4)	67.32 (21.18)		59.74 (19.73)		52.12 (24.66)	
**Work status**							
Full-time	185 (88.5)	68.08 (21.05)	*t* = 1.29 (0.24)^a^	60.45 (19.94)	***t*** **=** **3.45 (0.00)^a^**	54.77 (23.04)	*t* = 0.10 (0.83)^a^
Part-time	16 (7.7)	60.93 (22.96)		52.86 (20.53)		54.16 (26.70)	
**Work shifts**							
Day shift	87 (41.6)	62.97 (21.812)	***f*** **=** **4.94 (0.00)^b^**	56.71 (19.29)	***f*** **=** **3.85 (0.00)^b^**	52.95 (25.24)	*f* = 0.61 (0.62)^**b**^
Night shift	26 (12.4)	64.743 (20.79)		55.58 (19.20)		58.23 (20.19)	
Rotating day and night	93 (44.5)	72.53 (20.09)		64.13 (20.48)		55.60 (21.94)	
**Work hours per week**							
<45 h.	40 (19.1)	54.06 (22.35)	***f*** **=** **7.33 (<0.001)^b^**	48.76 (21.30)	***f*** **=** **6.82 (<0.001)^b^**	51.85 (23.90)	*f* = 2.01 (0.16)^**b**^
45–55 h	128 (61.2)	70.21 (19.88)		61.38 (19.20)		54.30 (23.32)	
56–65 h	26 (12.4)	72.91 (21.99)		64.01 (17.49)		54.80 (20.29)	
More than 65 h	12 (5.7)	71.87 (14.33)		72.61 (16.63)		70.13 (21.38)	
**Do you feel at risk of being infected by (COVID-19)?**							
Yes	170 (81.3)	67.57 (21.13)	*t* = 0.43 (0.91)^a^	59.69 (20.05)	*t* = 0.01 (0.29)^a^	54.22 (23.10)	*t* = −0.57 (0.44)^a^
No	36 (17.2)	65.85 (24.51)		59.65 (21.98)		56.68 (25.13)	
**Have you tested positive for COVID-19?**							
Yes	72 (34.4)	69.61 (21.17)	*t* = 1.15 (0.23)^a^	62.2 (19.94)	***t*** **=** **1.53 (0.05)^a^**	60.20 (23.99)	***t*** **=** **2.57 (0.01)^a^**
No	135 (64.6)	65.95 (21.89)		58.32 (20.43)		51.53 (22.61)	
**Have you ever been quarantined?**							
Yes	86 (41.1)	67.53 (21.00)	*t* = 0.22 (0.97)^a^	59.95(20.66)	*t* = 0.18 (0.29)^a^	59.56 (24.94)	***t*** **=** **2.59 (0.01)^a^**
No	120 (57.4)	66.84 (22.22)		59.42 (20.18)		51.06 (21.74)	
**Was anyone in your family found to be COVID-19 positive?**							
Yes	157 (75.1)	68.57 (20.84)	*t* = 1.59 (0.16)^a^	59.99 (19.98)	*t* = 0.36 (0.46)^a^	54.80 (24.19)	*t* = 0.27 (0.78)^a^
No	50 (23.9)	63.00 (23.78)		58.78 (21.45)		53.75 (20.98)	
**During your interaction with COVID-19 patients, were you wearing adequate PPE?**							
Yes	166 (79.4)	69.10 (21.24)	***t*** **=** **2.09 (0.02)^a^**	61.00 (20.37)	*t* = 1.52 (0.10)^a^	54.47 (23.96)	*t* = −0.46 (0.60)^a^
No	39 (18.7)	61.21 (20.83)		55.55 (18.71)		56.41 (19.91)	
**Did your shift time change during the pandemic?**							
Yes	157 (75.1)	69.66 (21.62)	***t*** **=** **2.91 (0.00)^a^**	61.37 (20.39)	***t*** **=** **2.12 (0.02)^a^**	56.02 (23.16)	*t* = 1.61 (0.15)^a^
No	50 (23.9)	59.583 (20.13)		54.44 (19.28)		49.91 (23.83)	
**Did your work hours change during the pandemic?**							
Yes	156 (74.6)	69.89 (21.64)	***t*** **=** **3.16 (0.00)^a^**	61.27 (20.61)	***t*** **=** **1.96 (0.01)^a^**	55.10 (23.43)	*t* = 0.59 (0.65)^a^
No	51 (24.4)	59.06 (19.80)		54.87 (18.71)		52.85 (23.48)	

*Bold values indicates significance at the 0.05 level*.

a *t-test*,

b *ANOVA*.

## Discussion

The study findings demonstrate considerable levels of burnout among frontline healthcare workers during the COVID-19 pandemic. Our result is consistent with several international studies conducted to investigate the psychological effect of COVID-19 and other infectious disease epidemics on burnout among frontline HCWs, as they encounter tremendous pressure to provide timely care (37–42). Indeed, rapid decision-making is key to proper COVID-19 diagnoses, isolation, and successful treatment, which in turn increases the burden on HCWs to establish quality healthcare (41). In India, about half of the respondents (52%) had pandemic-related burnout (43). In Iran, 53% experienced high levels of burnout (28). In Saudi Arabia's early months of the pandemic (June-August, 2020), the prevalence of burnout among HCWs was 75% (44). This current study (October 2020) found that HCWs are scoring the highest burnout on personal and work-related burnout, while patient-related burnout is the lowest. This is similar to a recent study done in Denmark that indicated that patients may only have a minor role in burnout among healthcare workers (45).

Regarding the association between sociodemographic and burnout the study found that age was not significantly associated with burnout in all three burnout scales, while marital status, nationality, and years of work experience were significant factors associated with patient-related burnout. These findings are consistent with a study by Chemali et al. (17) that showed inconsistent relation between burnout, age, and years of experience. In addition, Barello et al. (29) suggest that some of the critical factors that might trigger burnout are the decreased social support, and the insufficient material and human resources.

Few studies reported that older age and more work experience were positively associated with burnout and psychological stress (46–50). Whereas other studies found that younger HCWs were more vulnerable to psychological disorders and older adults were less likely to develop burnout during pandemics (51, 52).

In terms of gender, females had significantly higher burnout level than males in both personal and work-related scales. Although this relationship is inconsistent in the literature, our finding was similar to studies that reported females at increased risk for burnout (38, 39, 53, 54). In contrast, other studies reported that the male gender is a predictor of depersonalization and is at higher risk of burnout (9, 55, 56). Few other studies reported no association at all between burnout and gender, such as a study in Turkey among medical students by Sevencan et al. and a study by Amiri in Iran among primary care physicians (9, 56).

Among HCWs in this study, nurses were found to be at greater risk of high burnout across work and patient-related scales. This finding is consistent with the results of previous studies in earlier pandemics (6, 7, 40). During the SARS epidemic, a study carried out among healthcare professionals in emergency departments showed that nurses were more likely to experience behavioral disengagement and develop distress than other healthcare professionals (49). Lai et al. (57) assessed mental health outcomes in frontline healthcare professionals during the COVID-19 pandemic in China. Their findings showed that frontline nurses caring for COVID-19 patients had a higher level of mental health disorders because of their frequent and close contact with patients and increased number of working hours (57). On the other hand, few studies reported that physicians were the most exposed to burnout amongst all healthcare professionals (58, 59).

Furthermore, irregular working shifting schedule was found to be positively related to burnout during the pandemic. A study among medical staff who work night shifts found them to be more vulnerable to psychological distress (17, 60, 61). Another study among nurses in a university hospital in Egypt, reported that working in a night shift and the number of shifts were predictors for high levels of burnout (53). Another study among Turkish nurses found that working in night shifts was more positively associated with burnout than working day shifts or occasional night shifts (60). The higher level of burnout among night shift nurses was attributed to the shortage of nurses working at night shifts, causing unbalanced nurse-to-patient ratios (61). Also, sleep deprivation is a well-reported causative factor for clinical burnout (62).

In contrary to studies suggesting that inadequate PPE can be a considered a principal stressor increasing the burnout (19, 63), our findings showed that those wearing adequate PPE while interacting with COVID-19 patients had higher personal burnout. As frontline HCWs are the first responders for COVID-19 patients, they can be physically and mentally exhausted during their prolonged wear of PPE given its limitations of comfort and reduced ability to communicate. Particular features of PPE can impose physiological and personal burden especially if accompanied with long work hours with few breaks for self-care, nutrition, and hydration (64). In addition, other factors such as obesity and respiratory conditions can exacerbate the PPE burden, and could cause heat stress, skin irritation, headache and dizziness which compromise HCWs wellbeing and patient safety (64, 65).

Further studies found elevated levels of burnout among HCWs working in a COVID-19 unit, those with low self-confidence in self-protection, those who reported uncertainty about future availability of PPE, and those who were unsure whether the type of PPE provided was appropriate for their role (66–68). In addition, the possibility of developing burnout symptoms increased two folds among physicians in Egypt who needed to buy their own PPE (69). Yet, the current study found that perceived threat for exposure to COVID-19 was not significantly related to any of the burnout scales, which is inconsistent with other studies (29).

As the work nature of HCWs is challenging, they are also a vulnerable group of which their mental health and wellbeing must be safeguarded. Earlier studies have indicated that nurses with high sources of social support reported less burnout (70, 71). However, although social and family support is important in coping, HCWs are cautious when spending time with their close family members due to the fear of spreading infection. In addition, many HCWs are reluctant to reveal their challenges and difficulties even when encountering significant psychological distress (72). Reducing the stigma of impaired mental health and promoting support and sharing among colleagues in the work environment can foster help-seeking attitudes and behaviors (73). For example, regular check-ins by frontline supervisors to support staff and assess their symptoms or concerns is a potential method to identify early signs of burnout. In addition, administrative control should be more effective by instituting policies and procedures for workload, workhours, and breaks especially among HCWs working in the ICU. Furthermore, a mechanism for timely reporting of burnout symptoms without fear of judgement or penalty can reinforce care for HCWs.

This study provides a significant addition to the literature on frontline HCWs burnout during the COVID-19 pandemic in the personal, work, and patient-related scales using a validated assessment tool. However, some of the encountered limitations include the limitation of the questionnaire to identify the cause of the burnout, and what could be ways to mitigate personal, work, or patient-related burnout among HCW. In addition, the nature of the study's cross-sectional design is a limitation, as causality between variables cannot be concluded. Also, there might be a risk of selection bias as the questionnaire was distributed online, and thus, did not reach HCWs who were busy or offline during the e-distribution period. Moreover, frontline HCWs experiencing a high level of burnout may be more interested in filling out the survey, making the results overestimate. However, the study builds on current literature to add an extensive assessment about HCW's factors that are associated with each burnout sub-scale, providing a holistic understanding of factors associated with frontline HCWs psychological health.

There is an urgent need to inform policymakers about this critical situation and recommend applicable and appropriate burnout prevention for the healthcare force. Although HCWs heavily rely on the training and equipment provided by their organizations, managerial support and effective leadership must be contributed to avoid and mitigate HCWs adverse psychological outcomes (73). Policy-makers need to adopt interventions to promote a healthy work environment and prevent burnout among HCWs during pandemics and emergencies.

## Conclusions

Frontline HCWs in Saudi Arabia face considerable burnout during the COVID-19 pandemic. Several sociodemographic and occupational factors have contributed to increasing HCWs burnout levels during the pandemic. Findings emphasize the need for urgent strategies on the individual and organizational level to discuss burnout factors and invest in psychological interventions that reduce the risk of burnout and promote HCWs wellness in facing prolonged pandemics.

## Data Availability Statement

The raw data supporting the conclusions of this article will be made available by the authors, without undue reservation.

## Ethics Statement

The studies involving human participants were reviewed and approved by the Institutional Research Board of Imam Abdulrahman Bin Faisal University: IRB# PGS-2021-03-167. The patients/participants provided their written informed consent to participate in this study.

## Author Contributions

All authors listed have made a substantial, direct, and intellectual contribution to the work and approved it for publication.

## Conflict of Interest

The authors declare that the research was conducted in the absence of any commercial or financial relationships that could be construed as a potentialconflict of interest.

## Publisher's Note

All claims expressed in this article are solely those of the authors and do not necessarily represent those of their affiliated organizations, or those of the publisher, the editors and the reviewers. Any product that may be evaluated in this article, or claim that may be made by its manufacturer, is not guaranteed or endorsed by the publisher.
