# Supplementary material for: Sociodemographic and Occupational Factors Associated With Burnout: A Study Among Frontline Healthcare Workers During the COVID-19 Pandemic
Source: Front Public Health. 2022 Mar 9;10:854687. doi: 10.3389/fpubh.2022.854687 (PMC8959574; doi:10.3389/fpubh.2022.854687)

***Supplementary file***

***Table 1: Multiple Comparisons: Post-hoc tests (HCW Profession)***

***Dependent variable: work-related burnout***


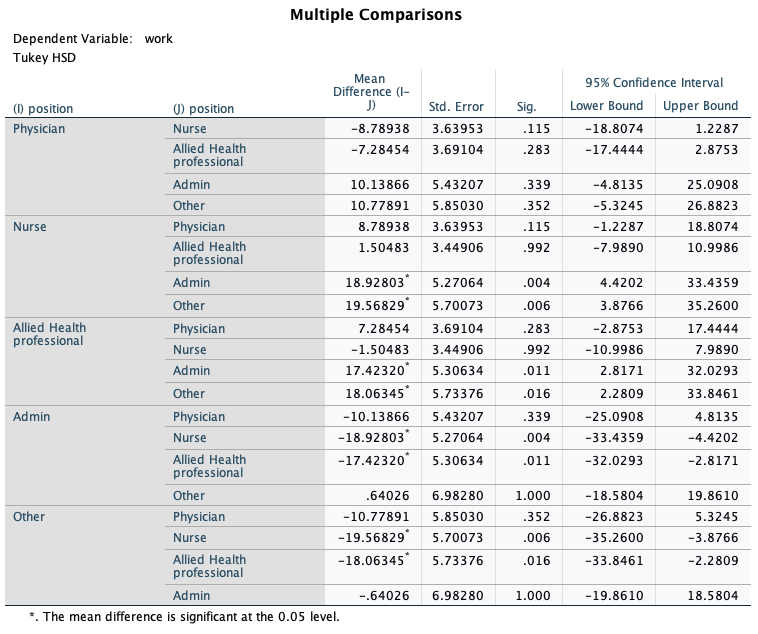

Supplement: Supplementary file 1 [file Table_1.docx]
